# Place Aversion by Morphine in Offspring Born of Female Morphine Administered Wistar Rats

**Published:** 2011

**Authors:** Manizheh Karami, Mohammad Reza Zarrindast

**Affiliations:** a *Department of Biology, Faculty of Basic Sciences, Shahed University, Tehran, Iran.*; b *Basic Sciences Research Center, Shahed University, Tehran, Iran.*; c *Department of Pharmacology, School of Medicine, Tehran University of Medical Sciences, Tehran, Iran.*

**Keywords:** Morphine, Sex-dependent, Place conditioning, Aversion, Pregnancy, Offspring (rat)

## Abstract

This research was designed to study sexual differences in place conditioning induced by morphine in offspring born of female Wistar rats mated with drug-naïve males. Mothers were exposed to morphine during the 14^th^-16^th^ days of gestational. Control dams were simply saline-injected. Female and male virgin offspring born of morphine-treated or saline-treated mothers were separately housed until become fully matured. A 3-day schedule of an unbiased conditioning procedure was used to the induce conditioning to morphine (2.5-7.5 mg/Kg, SC) in the offspring. According to the results, female offspring born of saline-administered mothers were morphine place-conditioned at lower doses of opioid (2.5 mg/Kg) in comparison to the males. An increase in locomotor activity in the females at 7.5 mg/Kg of opioid was also revealed. In contrast, administration of morphine (2.5-7.5 mg/Kg, SC), induced a significant aversion in either sexes of offspring born of morphine-exposed mothers. Moreover, female offspring of this category acquired more pronounced aversion at higher doses of morphine than males. In addition, a significant morphine-dose effect (7.5 mg/Kg, SC) on locomotor activity of these females’ offspring was observed. This study may highlight sex differences in conditioning effects induced by morphine between offspring derived of morphine-treated mothers and those of saline-treated.

## Introduction

Little evidence notifies that the administration of morphine in adult male rats adversely affected pregnancy outcome after mating with opioid-naïve females. This evidence further shows that pseudo-pregnancies obviously increased in females mated with morphine treated males (1). Findings also indicate that the exposure of male rodents to opiate leads to adverse effects on the development of offspring resulting of mating of these animals with drug-naïve females (2-5). Moreover, it has been postulated (6) that the positive reinforcing effects of morphine after repeated exposure is retained for at least one month, suggesting a type of behavioral sensitization to the drug of abuse (7, 8). However, there is no evidence to clarify if exposure of female rodents to morphine in a task of place conditioning through their first pregnancy, has an influence on the offspring› conditioning to the drug after they being matured.

In the present study, we decided to survey on conditioning to morphine in the offspring born of mothers suffering from repeated injections of morphine through 14^th^-16^th^ days of gestational to examine the potency of the prenatally morphine exposure to induce aversion which is possibly related to the opioid addiction. In view of evidence conveying that sex plays an important role in morphine conditioning (1, 10), we further worked on sex-differences in morphine conditioning of prenatally morphine exposed offspring. As another behavioral sensitization parameter of locomotor activity, an important factor in the development and maintenance of addiction was studied by our laboratory.

## Experimental

*Subjects*

Adult male and female rats (weighing 200-220 g, Wistar, Pasteur Institute of Iran, Tehran, Iran) were housed in standard plastic cages in groups of 2 in a controlled colony room (temperature 21 ± 2°C). They were maintained on a 12 h light/dark cycle with food and water *ad libitum*. Females and males were coupled in groups of 2 for mating. After observation of vaginal plugs, females were isolated and injected morphine (2.5-7.5 mg/Kg, SC), using the procedure of conditioning during 14^th^-16^th^ days of gestational. Control pregnant females were simply injected saline in the task. The pups born of the saline-injected or morphine-injected mothers were weaned at the age of 20 days. They were then isolated based on gender and prenatally exposure to morphine or saline. When offspring got matured, they were conditioned to morphine (2.5-7.5 mg/Kg, SC) using a 3-day schedule of an unbiased paradigm (10, 11, 12). The experiments were carried out during the light phase of the cycle. A total of 8 animals per group were used and tested only once. At the end of the experiments, the rats were killed with overdose of chloroform. The experiments were conducted according to a protocol approved by Committee of Ethics of Shahed University (Document No: 4-46/1379).

*Drugs*

Morphine sulphate (TEMED, Co., Tehran, Iran) was prepared fresh in sterile 0.9% NaCl solution. All injections were made subcutaneously (SC) and the vehicle was saline (1 mL/Kg) in all experiments.

*Apparatus*

A two compartment conditioned place preference apparatus (30 X 60 X 30 cm) was used in these experiments. Place conditioning was performed using an unbiased procedure, design of which was previously described (10, 11, 12); the apparatus was divided into two equal-sized compartments. In the middle of the apparatus, a removable wall was designed. Both compartments were colored white but differently striped black (vertical vs. horizontal). To provide the tactile difference between the compartments, one of the compartments had a smooth floor, while the other one had a nylon white mesh floor.

*Pre-conditioning (familiarization)*

Animals on day 1 received one habituation session. They were placed in the middle line of apparatus and had free access to the entire apparatus for 15 min, while the removable wall was raised 12 cm above the floor. The time spent by rats in each compartment was recorded by an observer who was blind to the results. None of the groups displayed a significant preference for one of the compartments, confirming that this procedure is unbiased.

*Conditioning*

This phase consisting of 6 sessions; 3-saline and 3-drug pairings was started on day 2 and lasted until day 4 during which the animals were daily injected drug or saline with a 6 h interval in a counterbalanced manner. Drug administration in conditioning phase was carried out during the light phase of a 12 h light/dark cycle. Control groups received saline (1 mL/Kg) twice a day with a 6 h interval. The duration of all conditioning sessions was 45 min. During these sessions, the removable wall was inserted along the seam separating the compartments. For each drug dose, 8 animals were used for half of which drug administration was paired with one compartment, while for the other half it was paired with the other (10, 13, 14). 

*Testing (Post-conditioning)*

Test sessions were carried out on 5^th^ day in a drug-free state. Each animal was tested only once. For testing, the removable wall was raised 12 cm above the floor and each of the uninjected animals was allowed free access to both compartments of the apparatus for 15 min. The time spent in the drug-paired compartment on testing day minus that of spent in the same side on day of familiarization representing the score of change in place preference (in sec) was calculated and expressed as mean ± SEM.

*Coupling of adult wistar rats and offspring housing*

Female and male Wistar rats were coupled in groups of two. All females were first-time pregnant. The female rats were isolated after observation of plug vaginal and maintained on a 12 h light/dark cycle with food and water *ad libitum*. Pregnant rats were injected morphine once a day (2.5-7.5 mg/Kg, SC) through the 14^th^-16^th ^days of their gestation period using the task designed for conditioning to morphine. Control groups were simply injected saline in the task. The Pups were fully cared until become weaned. They were then isolated based on gender and prenatally morphine-treatment or saline-treatment. They were maintained in same-sexes groups of two on a 12 h light/dark cycle with food and water *ad libitum *to become fully matured (8 weeks). When offspring got matured they were conditioned to morphine (2.5-7.5 mg/Kg, SC) using a 3-day schedule of an unbiased paradigm (10, 11, 12). A total of 8 pups were assigned to form an experimental group. A random selection of pups born of mothers gestationally received morphine was followed to eliminate the possible prenatal dose-effect of morphine.

*Induction and assessment of morphine place conditioning*

The effects of subcutaneous (SC) administration of morphine (2.5, 5, and 7.5 mg/Kg) on the induction of a conditioned place preference in animals were determined. Each morphine dose or saline was injected in a 3-day schedule of conditioning as described in detail in the experimental. The time spent in the drug-paired side on testing day minus that of spent in the same side on day of familiarization was calculated to assess the conditioned place preference induction. Each drug dose was tested once in 8 animals in a morphine-free state.

*Measurement of drug-treatment effects on locomotor activity*

Locomotor testing was carried out on the 5^th^ day of the task for rats which received 3-morphine pairing, using the conditioned place preference apparatus. To measure the locomotor activity according to a design previously described (12), the ground area of the conditioned place preference compartments was divided into 4 equal-sized squares. Locomotion was thus measured by an observer who was unfamiliar to the results as crossing from one square to another. Results were expressed as counts obtained per animals over a 15 min testing. This parameter was measured so that the evidence expressing that change in locomotion be observed in presence of stimuli which were previously associated with the positive reinforcers (15).

*Measuring the effects of drug treatments on body-weight*

The body-weight of experimental animals was recorded prior to the conditioning procedure (on familiarization day) and thereafter, in conditioning phase (4^th ^day) prior to the administration of drug. The parameter was expressed as the change in body-weight (in g) on 4^th^ day to that of the pre-conditioning (data were not shown).

*Criteria used to evaluate the maternal-littermates interactions at birth or dams*’ *care patterns*

The role of parents in the early development of their offspring by providing pups with nutrients, heat and protection was studied to notify the maternal-littermates interactions at birth. Furthermore, the development of handling stress vulnerability in offspring was measured to evaluate the dams› care patterns.

*Statistics*

One-way or two-way analysis of variance (ANOVA) followed by Tukey-Kramer or Newman keul’s multiple comparison tests were used to determine whether statistically significant differences were generated between treatments upon drug-induced response compared to the control groups. The p- values less than 0.05 were considered as significant.

## Results and Discussion

*Induction and assessment of morphine place conditioning produced in male and female offspring born of both morphine-treated and saline-treated dams*

**Figure 1:**
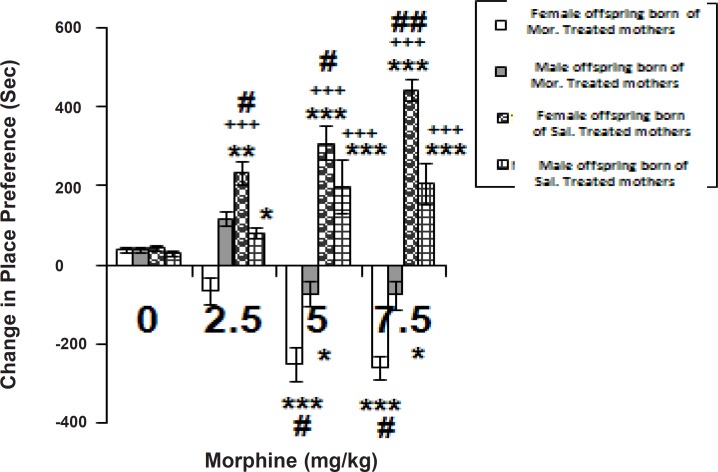
Place conditioning by morphine in two sexes of offspring born of saline-injected or morphine-injected females through gestation. Morphine (2.5-7.5 mg/Kg) or saline (1 mL/Kg) was given subcutaneously (SC) in a 3-day schedule of an unbiased conditioning paradigm. Control groups were simply injected saline (1 mL/Kg, SC), twice daily for 3 days. The data are expressed as mean of change in place preference ± SEM. Change in place preference is defined as the time spent in the drug-paired place on day of testing minus that of spent in the same side pre-conditioning. Tukey-Kramer post-hoc analysis showed the differences as follows: *p < 0.05, **p < 0.01 and ***p < 0.001 difference to respective control groups. +++p < 0.001 difference between the same sexes of offspring in two categories (Females in morphine-treated category vs. Females in saline-treated one or Males in morphine-treated category vs. Males in saline-treated one) # p < 0.05, and ## p < 0.01 difference between the opposite sexes (Females vs. Males) in each category

Figure 1 shows the response to morphine (2.5-7.5 mg/Kg, SC) in the place conditioning paradigm in male and female offspring born of both morphine-treated and saline-treated pregnant Wistar rats.

We used two-way ANOVA followed by Tukey-Kramer multiple comparison tests to clearly compare the response to morphine between the sex groups of offspring of both categories. Two-way ANOVA revealed differences at a significant level [Factor dose (3, 56) = 1.856, p < 0.0001; Factor sex (1, 56) = 255.74, p < 0.0001; Factor sex x dose (3, 56) = 40.8028, p < 0.0001]. More analysis using Tukey-Kramer multiple comparison tests indicated that morphine (2.5, 5, and 7.5 mg/Kg, SC), during conditioning, induced a significant conditioned place preference in male and female offspring born of saline-injected mothers in respect to control. [F (3, 28) = 31.565, p < 0.0001 in females, and F (3, 28) = 21.939, p < 0.0001 in the other sex]. Further analysis showed that the female offspring acquired conditioned place preference at lower doses of morphine (2.5 mg/Kg, Figure 1) than the males. Moreover, the response-induced by opioid at the higher doses (5-7.5 mg/Kg) was more pronounced in the females than the males. As data shows, the rats which received saline (1 mL/Kg, SC) twice per day (control groups), during conditioning, exhibited no preference for two compartments of the conditioned place preference apparatus on the day of testing.

Figure 1 also shows the response to morphine (2.5-7.5 mg/Kg, SC) in the place conditioning task in male and female offspring born of morphine-treated mothers. During the conditioning, administration of morphine (2.5, 5, and 7.5 mg/Kg, SC) induced a significant response in both sexes of this category in respect to the controls [F (3, 28) = 14.366, p < 0.0001 in females, and F (3, 28) = 15.859, p < 0.0001 in the other sex]. Post tests manifested that the female offspring acquired more pronounced aversion at the higher doses of morphine (5-7.5 mg/Kg, Figure 1) than the males. Based on the results, morphine sex-dependently induced the place conditioning in offspring born of mothers of both categories and also the fact that the opioid induced a negative response (aversion) in offspring born of morphine-treated mothers. In contrast, the response to the drug was positive in those born of saline-injected dams.

*Effects of morphine on locomotor activity of male and female offspring born of both morphine-treated and saline-treated dams*

**Figure 2:**
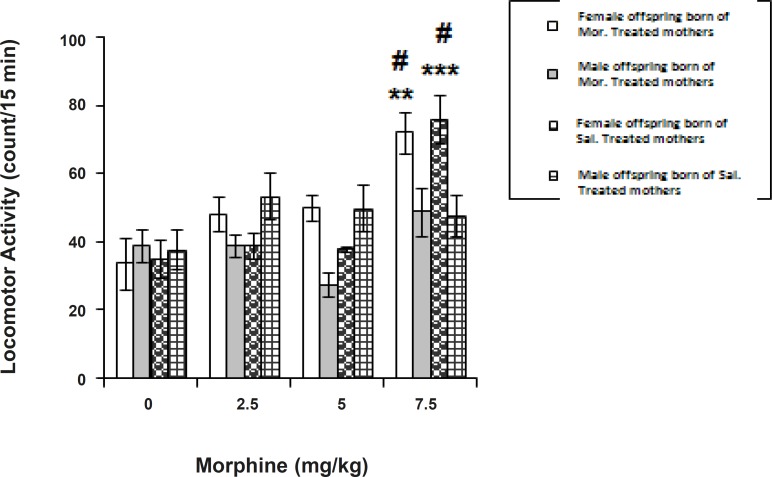
Effect of morphine on locomotor activity of offspring born of saline-treated or morphine-treated offspring through gestation Morphine (2.5-7.5 mg/Kg) or saline (1 mL/Kg) was given subcutaneously (SC) in a 3-day schedule of an unbiased conditioning paradigm. Control groups received saline (1 mL/Kg, SC) twice daily for 3 days. The locomotor activity of conditioned animals during a period of 45 min is assessed as described in Experimental. The data are expressed as mean of counts obtained per animal over a 15 min testing ± SEM. Tukey-Kramer post-hoc analysis showed the differences as follows: **p < 0.01, and ***p < 0.001 difference to respective control groups # p < 0.05 difference between the opposite sexes (Females vs. Males) in the categories

Figure 2 shows the effect of morphine (2.5-7.5 mg/Kg, SC) on locomotor activity of conditioned offspring born of saline-treated or morphine-treated dams. In order to compare the response to morphine between respective sexes of offspring of both categories, a two-way ANOVA followed by Tukey-Kramer multiple comparison test was used which indicated the dose differences at a significant level [Factor dose (3, 56) = 17.811, p < 0.0001; Factor sex (1, 56) = 170.959, p < 0.001; Factor sex x dose (3, 56) = 197.452, p < 0.0001]. According to the analysis, morphine induced hyperactivity in female offspring derived of both categories of mothers.

One-way ANOVA revealed a significant morphine dose factor in the female offspring born of the saline-injected category [F (3, 28) = 15.194, p < 0.0001, in females, and F (3, 28) = 1.083, p > 0.05 in males]. More analysis indicated an increase in locomotor activity in the females at 7.5 mg/Kg of opioid.

One-way ANOVA further revealed a significant morphine dose factor in the female group born of the morphine-treated category [F (3, 28) = 6.086, p < 0.01, in the females, and F (3, 28) = 2.686, p > 0.05 in the males]. Further analysis indicated an increase in locomotor activity in females at 7.5 mg/Kg of opioid.

*Measurement of maternal-littermates interactions at birth or dams› care patterns*

Maternal behavior including maternal-littermate interactions at birth or dams› care patterns showed no difference between two categories of mothers (saline-treated group vs morphine-treated group) as the finding showed (F (1, 28) = 1.083, p > 0.05).

Based on the results, subcutaneous (SC) injections of morphine (2.5, 5, and 7.5 mg/Kg) induced a significant conditioned place preference in both sexes of offspring born of saline-treated mothers. This category of mothers was simply administered saline through their gestation period. Female offspring derived of this category acquired conditioned place preference at lower doses of morphine than the males (2.5 mg/Kg, Figure 1). Moreover, the response was more pronounced in females than the males. This fact indicates that female offspring born of saline-injected mothers is more sensitive to the rewarding effects of morphine. The obtained data are in accordance with the previous achievement (10). On the other hand, morphine differently induced a significant aversion in offspring born of morphine-treated female Wistar rats. Morphine response was significantly more pronounced in the female offspring of this category than the other sex. It can be suggested that early exposure to morphine in these groups as a result of maternal exposure to the opioid in the conditioning task during gestation, may result in alterations in the behavioral responsiveness of the animals to the conditioning effects of the drug. 

A report by Riley and Vathy (2006) showed that mid to late gestational morphine exposure does not alter the rewarding properties of morphine in adult male rats (16). They used much more doses of morphine in the CPP paradigm, however, due to the failure in showing that the CPP of morphine was dose-dependent, they demonstrated that the prenatally morphine exposure has no effect later in the rewarding through morphine. In fact, as the main finding, they proposed that only one group of the offspring, those born of morphine-treated with low dose morphine (0.1 mg/Kg, SC), shows a significant CPP. The different conclusions between ours and those of Riley and Vathy (2006) could be interpreted by considering both the way of morphine exposure through the gestational period and the selection of marginal doses of morphine to induce CPP as well.

In a recent study by Wu *et al. *(17), dextromethorphan co-administered with morphine during pregnancy to survey on morphine-induced reward and behavioral sensitization in male offspring born of dextromethorphan-morphine-exposed mothers. They revealed that the male offspring born of chronic morphine-treated female rats are more vulnerable to morphine-induced reward and behavioral sensitization. They further indicated that the administration of a low dose of morphine (1 mg/Kg, IP) in the male offspring causes an increase in the dopamine and serotonin turnover rates in the nucleus accumbens. This work and ours may clearly explain that morphine administrations during pregnancy provide the potency of the opioid to induce addiction in the prenatally morphine-exposed offspring. However, the mechanism of changes in the opioid system in the offspring as a result of prenatally exposure to morphine remains to be determined.

Maternal behavior showed no difference statistically between the two categories of mothers, as the measurement of maternal-littermate interactions at birth or dams› care patterns, indicating that the maternal exposure to the opioid, 3-sessions, during the gestation, failed to induce changes in maternal behavior. In the contrary, the inhibition of maternal behavior by morphine has been recorded in morphine-treated dams (18, 19, 20). The facts may express that the behavior is most probably dependent to the dose of the drug. So, larger doses of the opioid and/or more session of exposure to the morphine through gestationally treatment might be required to induce a change of maternal behavior at a significant level.

We examined another behavioral parameter, behavioral sensitization of locomotor activity, which has been viewed as an important factor in the development and maintenance of morphine reward (19, 20). In this study, sex-differences in locomotor activity were recorded due to the conditioning to morphine in both categories of offspring born of saline- or morphine- gestationally treated rats. The female offspring of both categories showed a significant response at 7.5 mg/Kg of the opioid, an effect which is not correlated with the magnitude of the conditioning induced through morphine in these offsprings as conditioned female offspring displayed higher locomotor activity in the drug-paired side during the preference testing. The results are in unity and corresponded in dose-effect with the the previous one (10). It has been argued that less activity in the drug-paired compartment allows subjects to prolong the contact with the drug-paired stimuli (23). Thus, the decrease, but not increase, in locomotor activity may cause a significant effect on the task of the place conditioning. Sex-differences in locomotor activity due to the morphine have also been shown by other researchers (24). These differences are attributed to the gonadal hormones in females (25). According to previous achievements, repeated administration of morphine results in augmentation of locomotor and/or stereotype behaviors (21, 22, 26), the effect which is named as «behavioral sensitization» and persisted for a long period after morphine withdrawal (27). This behavior was also presumed to be caused by the sensitization of mesolimbic dopaminergic pathway (28, 29). In contrast, present female offspring of both categories displayed higher locomotor activity in the drug-paired side during the preference testing, showing that prenatal morphine exposure may not cause further sensitization of the mesolimbic pathway. However, this fact reconfirms sex-differences in the effect of prenatally morphine exposure on locomotor activity in the CPP task. 

In the present study, no significant effects were shown via morphine at 2.5-7.5 mL/Kg on body weight in both sexes of all experimental animals, suggesting that the opioid at the doses used in the present study, does not affect feeding in the first generation of Wistar rats. Recent findings (10) indicated that the injections (3 pairings) of a high dose (10 mL/Kg) of morphine in Wistar rats facilitates the feeding in a way possibly dependent to the sex hormone by increasing the reward property of food (30). 

In a conclusion, differences can be observed across the effects of morphine in offspring derived from female Wistar rats administered morphine through their first pregnantation to those born of non-morphine treated rats. 
